# Extrastriatal changes in patients with *late-onset* glutaric aciduria type I highlight the risk of long-term neurotoxicity

**DOI:** 10.1186/s13023-017-0612-6

**Published:** 2017-04-24

**Authors:** Nikolas Boy, Jana Heringer, Renate Brackmann, Olaf Bodamer, Angelika Seitz, Stefan Kölker, Inga Harting

**Affiliations:** 1 Centre for Child and Adolescent Medicine, Clinic I, Division of Neuropaediatrics and Metabolic Medicine, University Hospital Heidelberg, Im Neuenheimer Feld 430, 69120 Heidelberg, Germany; 2 Department of Child and Adolescent Medicine, Klinikum Herford, Schwarzenmoorstrasse 70, 32049 Herford, Germany; 3 Division of Genetics and Genomics, Boston Children’s Hospital, Boston, MA USA; 4 Department of Neuroradiology, University Hospital Heidelberg, Im Neuenheimer Feld 400, 60120 Heidelberg, Germany

**Keywords:** Glutaric aciduria type I, Late-onset, Long-term disease course, Subependymal nodules, Frontotemporal hypoplasia, High excretor

## Abstract

**Background:**

Without neonatal initiation of treatment, 80–90% of patients with glutaric aciduria type 1 (GA1) develop striatal injury during the first six years of life resulting in a complex, predominantly dystonic movement disorder. Onset of motor symptoms may be *acute* following encephalopathic crisis or *insidious* without apparent crisis. Additionally, so-called *late-onset* GA1 has been described in single patients diagnosed after the age of 6 years. With the aim of better characterizing and understanding *late-onset* GA1 we analyzed clinical findings, biochemical phenotype, and MRI changes of eight *late-onset* patients and compared these to eight control patients over the age of 6 years with early diagnosis and start of treatment.

**Results:**

No *late-onset* or control patient had either dystonia or striatal lesions on MRI. All *late-onset* (8/8) patients were high excretors, but only four of eight control patients. Two of eight *late-onset* patients were diagnosed after the age of 60 years, presenting with dementia, tremor, and epilepsy, while six were diagnosed before the age of 30 years: Three were asymptomatic mothers identified by following a positive screening result in their newborns and three had non-specific general symptoms, one with additional mild neurological deficits. Frontotemporal hypoplasia and white matter changes were present in all eight and subependymal lesions in six *late-onset* patients. At comparable age a greater proportion of *late-onset* patients had (non-specific) clinical symptoms and possibly subependymal nodules compared to control patients, in particular in comparison to the four clinically and MR-wise asymptomatic low-excreting control patients.

**Conclusions:**

While clinical findings are non-specific, frontotemporal hypoplasia and subependymal nodules are characteristic MRI findings of *late-onset* GA1 and should trigger diagnostic investigation for this rare disease. Apart from their apparent non-susceptibility for striatal injury despite lack of treatment, patients with *late-on*s*et* GA1 are not categorically different from early treated control patients. Differences between *late-onset* patients and early treated control patients most likely reflect greater cumulative neurotoxicity in individuals remaining undiagnosed and untreated for years, even decades as well as the higher long-term risk of high excretors for intracerebral accumulation of neurotoxic metabolites compared to low excretors.

**Electronic supplementary material:**

The online version of this article (doi:10.1186/s13023-017-0612-6) contains supplementary material, which is available to authorized users.

## Background

In glutaric aciduria type 1 (GA1, OMIM#231670), a rare inherited metabolic disease, deficiency of glutaryl-CoA dehydrogenase results in accumulation of the putatively neurotoxic metabolites glutaric and 3-hydroxyglutaric acid (GA, 3-OH-GA) in body tissues, particularly within the brain. Two biochemical phenotypes, low and high excretors, have been defined based on urinary excretion of GA [1].

Without treatment the majority of patients develops an acute encephalopathic crisis during catabolic states, i.e. febrile illness, possibly triggered by vaccinations, or surgical procedures, during the first six years of life (“*acute-onset* type”), with acute striatal injury and subsequent complex movement disorder which may be best described as dystonia superimposed on axial muscular hypotonia [2]. In contrast, most patients diagnosed by newborn screening (NBS) and treated according to guideline recommendations since neonates, remain asymptomatic [3]. Two further clinical subtypes have been proposed: *insidious-* and *late-onset* types. Patients with *insidious-onset* (about one third of symptomatic patients [4]) develop a complex dystonic movement disorder due to striatal injury but *without* clinically apparent precipitating event [2–8].

The term *late-onset* has been used for patients diagnosed after the age of six years, who presented with - in comparison to patients with *acute* or *insidious* onset types - non-specific and sometimes longstanding symptoms and predominantly white matter changes on MRI [9–13]. Case reports on *late-onset* GA1 include four women, whose diagnostic work-up was initiated following their unaffected newborns’ positive NBS (*maternal GA1*), but not because of neurological presentation. Due to transplacental exchange, decreased concentrations of free maternal carnitine or increased maternal glutarylcarnitine can be detected by NBS in the non-affected neonate. Neonatal parameters normalize during the following weeks and confirmatory work-up of the non-affected neonate is negative which then triggers investigation of the mothers [14–16].

Current literature on *late-onset* GA1 only consists of case reports. With the aim of better characterizing and understanding *late-onset* GA1 we systematically evaluated clinical findings, biochemical phenotype (high/low excretor), and MRI changes in a sample of eight *late-onset* patients and compared these to eight control patients older than 6 years, who had been diagnosed and treated early.

## Methods

As part of our ongoing prospective study on long-term outcome of GA1 patients in Germany since 2002 [3, 17] we identified those patients who met diagnostic criteria of *late-onset* GA1 based on systematic evaluation of previously reported individuals with presumptive *late-onset* disease, namely (1) diagnosis after the age of 6 years, (2) absence of dystonic movement disorder, and (3) absence of striatal lesions on cerebral MRI. These *late-onset* patients were compared to control GA1 patients who (1) had been diagnosed and treated within the first three years of life, (2) had reached the age of at least 6 years, and had (3) neither clinical evidence of a dystonic movement disorder (4) nor striatal lesions on cerebral MRI.

Using these criteria we identified eight *late-onset* patients with a total of 22 MRI scans (age at diagnosis 8.5-71 years, age at MRI 8.5-73.8 years), including two patients previously published by our group [12] and a further patient whose initial case was presented in [18]. Eight control patients (age at diagnosis 8 days–36 months) with a total of 15 MRI scans performed between the ages of 6.2 and 22.1 years were identified for comparison. Initial findings of three of these have previously been reported as cases by us (c1 = case 2 (≤50 months), c2 = case 3 (≤36 months), c6 = case 4 (≤15 years) in [18]).

MRI scans were assessed for (a) frontotemporal hypoplasia, presence of abnormal (b) gray and/or (c) white matter signal [18], and (d) space-occupying lesions by an experienced paediatric neuroradiologist.

Clinical, biochemical and genetic patient characteristics are summarized in Table 1. Biochemical phenotype (low/high excretor) was classified based on urinary concentrations of GA (in mmol/mol creatinine) at time of diagnosis. Initial values were not available for the high-excreting patient 7, however, concentration on follow-up investigation was clearly elevated > 100 mmol GA/mol creatinine and thus within the range for high excretors [1]. It is known that urinary concentrations of GA and 3-OH GA remain elevated in high excretors despite some reduction with dietary treatment and do not decrease to the range of low excretors (<100 mmol/mol creatinine) [4, 19, 20].

**Table 1:** Clinical characteristics of *late-onset* type and control GA1 patients (listed according to age at first MRI)

	Age at dia-gnosis.	Gender (nation of origin)	Mode of diagnosis	Clinical symptoms until and/or at diagnosis	Clinical status at last follow-up visit (age in years)	Macro-cephaly	Biochem. phenotype: low/high excretor, GA and 3-OH-GA [mmol/mol Crea] at diagnosis unless otherwise stated	Mutation analysis of *GCDH* gene (chr. 19p13.2)	Resid. enzyme activity
Pat. #									
p1 ^a^	8.5 years	F (German)	targeted metab. scr.	nausea, vertigo	asympt. (17)	no	High GA 973 3-OH-GA 47	p.Arg128Gln/p.Glu414Lys	n.d.
p2 ^b^	15 years	M (German)	targeted metab. scr.	vertigo, headache, ↓motor balance, ↓fine mot. skills	Headaches (15)	yes (since infancy)	High strong elevation (no quantit.)	p.Arg88Cys,/p.Arg88Cys	<1%
p3	22 years	F (Syrian)	targeted metab. scr.	none; path. NBS of 1^st^ child (maternal GA1)	Headaches (22)	no	High GA 1742 3-OH-GA 124	p.Pro128Gln/p.Pro128Gln	n.d.
p4	29 years	F (German)	targeted metab. scr.	none; path. NBS of 2^nd^ child (maternal GA1)	headaches, coordination deficits, cognitive disability (40)	no	High GA 1527 3-OH-GA 94	p.Arg128Gln/Arg402Trp	n.d.
p5	18 years	F (German)	targeted metab. scr.	headache	asympt. (31)	no	High GA 1590, 3-OH-GA 133 (at last visit; n.d. at diagnosis)	p.Tyr74Thrfs^a^68/p.Arg132Gly	n.d.
p6	25 years	F (Turkish)	high risk scr.	none; mother of index patient	headaches, intermitt. vertigo (38)	yes (noted at diagn.)	High strong elevation (no quantit.)	n.d. (Glu365Lys/p.Glu365Lys detected in four other family members)	<1%
p7	71 year	M (German)	targeted metab. scr.	repetitive cerebral ischemia, intention tremor ≥62 years confusion,↓memory ≥ 65 years epilepsy ≥72 years	progressive dementia, tremor, epilepsy, dysdiadochokinesis, incontinence (73)	yes (noted at diagn.)	High GA 2149 (FU: 452) 3-OH-GA 99 on FU (n.d. at diagnosis)	p.Arg402Trp/p.Ala421Val	n.d.
p8 ^b^	66 years	M (German)	targeted metab. scr.	headache ≥35 years tremor ≥50 year seizures ≥54 years dementia, ↓speech ≥63 years	dementia, tremor, epilepsy, ↓speech, intermitt. orofac. dyskinesia, dysmetria, ↓fine motor skills (66):	yes (since infancy)	High GA 1595, 3-OH-GA 109	p.Arg383Cys/p.Arg383Cys	n.d.
Contr. #									
c1 ^c^	8 d	F (German/ Italian)	NBS	-	asympt. (12)	no	High GA 3695 3-OH-GA 77	p.Pro248Leu/p.Pro248Leu	1.5%
c2 ^c^	17 d	M (German)	NBS	-	asympt. (13)	no	High GA 4509 3-OH-GA 128	p.Arg313Trp (maternal origin)/no paternal mutation found	1%
c3	10 week	F (Swiss)	NBS/targeted metab. scr.	macrocephaly	asympt. (9)	no (init. yes)	Low GA 33; 3-OH-GA 17.5	p.Arg227Pro/p.Arg88Cys	n.d.
c4	10 d	M (German/ Indonesian)	NBS (younger sib. of c7)	-	asympt. (16)	no	Low GA 7; 3-OH-GA 25	p.Phe236Leu/p.Ser259Pro	3%
c5	13 m	F (German/ Mauretania)	high risk scr. (affected twin sister)	-	asympt. (11)	no	Low GA 6 (on FU at 2.5 years) 3-OH-GA 24 (on FU at 2.5 y)rs	p.Ala421.Val/p.Met405.Val	n.d.
c6 ^c^	pre-natal	M (German)	high risk scr. (younger sib. of c6)	-	asympt. (24)	yes	High GA >2.000 3-OH-GA 50	p.Gly185Ala/p.Cys176 Arg	n.d.
c7	3 m	M (German)	targeted metab. scr.	macrocephaly	asympt. (26)	yes	High GA 2670, 3-OH-GA elevated (no quant.)	p.Gly185Ala,/p.Cys176Arg	n.d.
c8	36 m	M (German/Indonesian)	targeted metab. scr.	global dev. delay (≥24 m)	vertigo (21)	no	Low GA normal (not detectable), 3-OH-GA 25	p.Phe236Leu, p.Ser259Pro	3%

*d* days, *dent.* dentate nucleus, *m* months, *mul*. multiple, *n.d.* not determined, *NBS* newborn screening, *pall.* pallidum, *scr.* screening, *sib.* sibling., *thal.* thalamus, *yrs*, years. ^a^ initial findings including MRIs at 8.5, 9.5, 10.4 years were reported in Harting et al. 2009 as case 5; ^b^ reported in [12]; ^c^ initial findings of c1, c2, and c6 were reported in Harting et al. 2009 (c1 = case 2 (≤50 months), c2 = case 3 (≤36 months), c6 = case 4 (≤15 years), [18])

Mutation analysis of the *GCDH* gene on chromosome 19p13.2 was performed using gDNA sequencing of all 11 exons with contiguous introns and was documented as changes of protein code (“p.” code) in all patients except patient 6, whose four other affected family members all carry the same mutation (p.Glu365Lys/p.Glu365Lys). In this patient as well as in p2, c1, c2, c4, and c8 measurement of residual enzyme activity in leukocytes had additionally been performed (Table 1).

With the exception of one *late-onset* patient without oral carnitine supplementation (p6), all control and *late-onset* patients were treated according to guideline recommendations [21]: All patients over the age of 6 years followed a protein-controlled diet using natural protein with a low lysine content and avoiding lysine-rich food and orally supplemented carnitine. Until the age of 6 years the early diagnosed control patients received combined metabolic therapy consisting of a low-lysine diet, carnitine supplementation, and a high-caloric, low- or no-protein emergency dietary regimen during episodes likely to induce catabolism.

## Results

### Diagnosis and clinical presentation

The eight *late-onset* patients were diagnosed between 8.5 and 71 years, five by targeted metabolic screening, two after diagnostic work-up of an initially abnormal NBS result of their newborns (“maternal GA1”, p3,4), and one female (p6) by high-risk family screening following diagnosis of her macrocephalic infant. According to the definition of Baric et al. [1], all eight *late-onset* patients were high excretors compared to four of the eight control patients (Table 1). Pathogenic variations (protein code changes “p.”) in the *GCDH* gene detected by molecular genetic analysis were different in all patients except for the siblings (c4/c8, c6/c7; Table 1).

Of the six *late-onset* patients diagnosed before the age of 30 years, the three patients identified via their infants (p3, p4, p6) were asymptomatic at diagnosis whereas the other three had non-specific, general symptoms like nausea, vertigo, and/or headaches, one (p2) with additional mild neurological deficits (impaired fine motor skills and motor balance). The two oldest patients (p7, p8) diagnosed at the age of 66 and 71 years presented with severe neurologic symptoms, namely dementia, tremor, and epilepsy. In p7 this had been preceded by several stroke-like episodes since the age of 61 years to which reduced strength of left upper extremity was attributed. Four patients (p2, p6-8) were macrocephalic and for two this had been documented since infancy (p2, p6). For six patients (p1-5, p8) various, minor febrile infections during infancy are known. In addition p4 had toxoplasmosis at age 19 years, p5 had two febrile seizures, and p3 had foot surgery at the age of 7 years. All patients fully recovered after each event and none developed symptoms of an acute an encephalopathic crisis or signs of an extrapyramidal movement disorder.

At last follow-up two of the six patients diagnosed before the age of 30 were asymptomatic; four had headaches, one with intermittent vertigo and one with coordination deficits and cognitive disability, but no evidence of cerebellar dysfunction. In the two patients diagnosed after the age of 60 years, dementia, tremor, and epilepsy persisted, in p8 with additional dysarthria, intermittent orofacial dyskinesia, dysmetria, and reduced fine motor skills. In p7 dementia was progressive and frequency of seizures increased despite metabolic and anticonvulsive treatment, moreover urinary and rectal incontinence developed (for detailed case report of p7 see Additional file 1).

Of the eight control patients four were diagnosed by NBS, one however without adequate diagnostic work-up and final diagnosis by targeted screening for macrocephaly at age of two months (c3). The other four patients were diagnosed before implementation of NBS by high-risk family screening due to an affected twin or older sibling (c5, c6) and by targeted screening for global developmental delay and macrocephaly (c7, c8). Except for unspecific intermittent vertigo in c8, all control patients were asymptomatic at last follow-up. Macrocephaly had resolved in c3 and persisted in two brothers (c6, c7), while none of the others were macrocephalic. No *late-onset* or control patient showed signs of a dystonic movement disorder at diagnosis or last follow-up visit.

### Frontotemporal hypoplasia

**Fig. 1:**
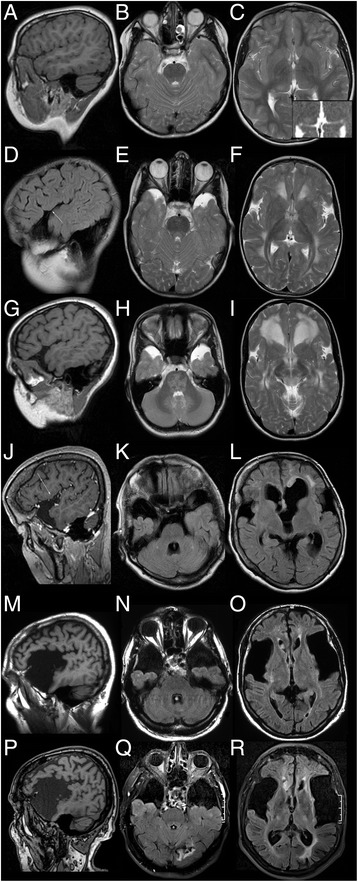
Normal frontotemporal anatomy and examples of frontotemporal hypoplasia. **a**-**c** Normal Sylvian fissure and anterior temporal CSF spaces in *control patient c2*. NB asymmetric thalamus with focal T2-hyperintensity and facilitated diffusion (inset: ADC map). **d**-**i** Mild hypoplasia in *late-onset patient p1* not extending dorsally beyond the pars opercularis (*arrow* in **d**) of the inferior frontal gyrus and remaining unchanged between first MRI at 8.6 years (**d**-**f**) and last follow-up MRI at 15.7 years (**g**-**i**). NB T2-hyperintensity of pontine white matter and dentate nucleus (H). **j**-**l** Moderate hypoplasia involving the subcentral gyrus in *late-onset patient p6* (*arrow* in **j**). NB subependymal nodules and focal, asymmetric *white* matter changes (**l**). **m**-**r** Widely open Sylvian fissure and massively widened anterior temporal CSF spaces in *late-onset patient p7* unchanged between MRIs at 61 and 73 years. NB large, subependymal, FLAIR-hyperintense nodule in right frontal horn (**o**, **r**; also Fig. 3). (*T1w:* **a**, **g**, **m**, **p**; *T1w + GAD:* **j**; *T2w:* **b**, **c**, **e**, **f**, **h**, **i**; *FLAIR:* K, **l**, **n**, **o**, **q**, **r**; *ADC:* inset in **c**)

All *late-onset* patients had frontotemporal hypoplasia (Table 2), which remained unchanged over a period of seven, eleven and twelve years in the three *late-onset* patients with serial MRIs (p1, p4, p7). Frontotemporal hypoplasia was mild in four patients (anterior Sylvian fissure not widened beyond the pars opercularis; p1, p3-5), moderate in two (involvement of subcentral gyrus; p2, p6), and most severe in the oldest patients with apparent expansion in p7 (Fig. 1).

**Table 2:** Neuroradiological characteristics of *late-onset* type and control GA1 patients (listed according to age at MRI)

	High/low excretor	Age at		Subependymal lesions			min. slice thickn.	wm changes	gm changes	Fronto-temp. hypoplasia
		diagnosis	at MRI	y/n	n=	max diam. [mm]				
Pat. #										
p1^a^	high	8.5 years	8.5 years	n	-	-	3.5 mm	y	pall., thal., dent.	y
			9.5 years	n	-	-	3.5 mm	y	pall., thal., dent.	y
			10.4 years	n	-	-	1 mm	y	pall., thal., dent.	y
			11.9 years	n	-	-	1 mm	y	pall., thal., dent.	y
			12.7 years	y	1	1.5	1 mm	y	pall., thal., dent.	y
			13.7 years	y	1	1.5	1 mm	y	pall., thal., dent.	y
			15.7 years	y	4	1.5	1 mm	y	pall., thal., dent.	y
p2^b^	high	15 years	14.7 years	y	mult.	3	2 mm	y	pall., dent.	y
p3	high	22 years	22.4 years	n	-	-	1 mm	y	dent.	y
p4	high	29 years	29.4 years	n	-	-	6 mm	y	dent.	y
			29.6 years	n	-	-	6 mm	y	dent.	y
			38.3 years	n	-	-	2 mm	y	dent.	y
			40.4 years	n	-	-	1 mm	y	dent.	y
p5	high	18 years	31.1 year	y	2	3	1 mm	y	dent.	y
p6	high	25 years	37.9 years	y	mult.	19	1.3 mm	y		y
p7	high	71 year	61.8 years	y	2	10	5 mm	y	right caudate (postischemic)	y
			65.1 year	y	2	10	5 mm	y	right caudate (postischemic)	y
			66.4 years	y	3	12	4 mm	y	right caudate (postischemic)	y
			70.2 years	y	4	13	4 mm	y	right caudate (postischemic)	y
			72.1 year	y	4	13	4 mm	y	right caudate (postischemic)	y
			73.8 years	y	5	13	1 mm	y	right caudate (postischemic)	y
p8^b^	high	66 years	66.3 years	y	1	17	6 mm	y		y
Controls #										
c1^c^	high	8 d	6.2 years	n	-	-	1 mm	y		y
c2^c^	high	17 d	7.2 years	n	-	-	1 mm	n	righ thal., dent.	n
			9.5 years	n	-	-	1 mm	n	righ thal., dent.	n
c3	low	10 week	8.7 years	n	-	-	1 mm	n		n
c4	low	10 d	10.6 years	n	-	-	1 mm	n		n
c5	low	13 m	11.0 year	n	-	-	1 mm	n		n
c6^c^	high	prenatal	11.0 year	n	-	-	3.6 mm	y	palllidum	y
			13 .0 year	n	-	-	3.6 mm	y	palllidum	y
			15.1 year	n	-	-	2 mm	y	palllidum	y
			19.1 year	n	-	-	1 mm	y	palllidum	y
c7	high	3 m	14.0 year	y	1	2	3.6 mm	y	palllidum	y
			16.0 year	y	2	2	3.6 mm	y	palllidum	y
			18.0 year	y	2	3	2 mm	y	palllidum	y
			22.1 year	y	2	3	1 mm	y	palllidum	y
c8	low	36 m	16.0 year	n	-	-	1 mm	n		n

*d* days, *dent.* dentate nucleus, *m* months, *mul*. multiple, *n.d.* not determined, *NBS* newborn screening, *pall.* pallidum, *scr.* screening, *sib.* sibling., *thal.* thalamus, *yrs*, years. ^a^ initial findings including MRIs at 8.5, 9.5, 10.4 years were reported in Harting et al. 2009 as case 5; ^b^ reported in [12]; ^c^ initial findings of c1, c2, and c6 were reported in Harting et al. 2009 (c1 = case 2 (≤50 months), c2 = case 3 (≤36 months), c6 = case 4 (≤15 years), [18])

Frontotemporal hypoplasia was less frequent in control patients: Five of eight control patients had no frontotemporal hypoplasia. The youngest control patient (c1) had mild, residual hypoplasia, and the two macrocephalic brothers (c6, c7) had continuously wide open Sylvian fissures, all three of these high-excretors.

### White matter changes

**Fig. 2:**
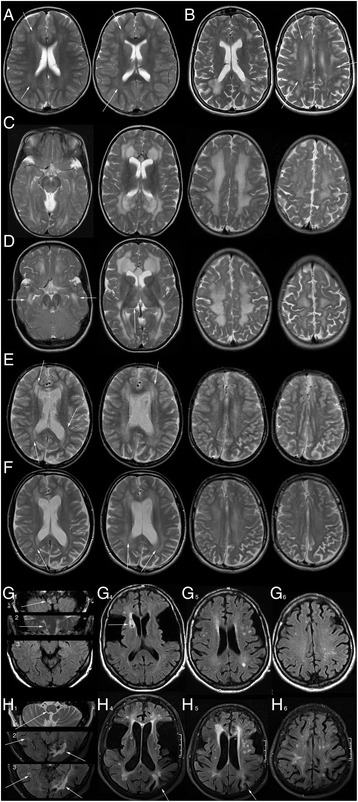
Examples of white and grey matter changes. **a** Mild, predominantly periventricular white matter changes (*arrows*) in *control patient c1*. **b** Focal and confluent, slightly asymmetrical white matter changes as well as mild periventricular white matter hyperintensity (*arrows*) in *late-onset patient p5*. **c**, **d** Extensive *white* matter changes in *late-onset patient p1* at 8.5 (**c**) and 15.7 years (**d**) with slight progression during follow-up (e.g. temporal, *arrows* **d**, compare with **c**). NB T2-hyperintensity of pallidum and medial thalamus is only depicted at follow-up (**d**, *arrow* to *right* thalamus) due to different slice angulation of examinations. **e**, **f** Extensive *white* matter changes in *control patient* c6 without progression between 11 (**e**) and 19 years (**f**) involving periventricular, lobar, and subcortical *white* matter. Pattern of involvement is similar to p1 with a rim of near normal signal (*arrows*) interspersed between changes of periventricular and more peripheral white matter. **g**, **h**. *White* and *grey* matter changes in *late-onset patient p7* at the age of 61 (**g**) and 73 years (**h**). At the age of 61 years there is a combination of mild, periventricular and multiple, *brighter white* matter hyperintensities undistinguishable from hypertensive white matter changes observed in non-GA1-patients at this age. Subacute ischemia of the *right* dorsolateral medulla oblongata (*arrows* in **g**_1,2_), which was the cause of the acute neurological presentation, retracts over time (*arrow* in **h**_1_) as does the pre-existing anterior lenticular and caudate defect (*arrow* in **g**_4_, compare with **h**_4_). By the age of 73 years, white matter changes have increased. Bilateral occipital (*arrows* in **h**_2,3_, compare with **g**_3_) and hemodynamic ischemia in the *left* parietal border zone (*arrows* in **g**_4,5_) have left defects and gliosis and there is overall volume loss with widening of ventricles and sulci. (*T2w:* **a**-**f**, **g**_2_, **h**_1_; *FLAIR:* **g**_1_, **g**_3–6_, **h**_2–6_)

All *late-onset* patients had white matter changes, predominantly of periventricular and lobar, frontal and parietal white matter. They were extensive in five (p1-4, p8; asymmetric in p8) and less extensive with additional multifocal, somewhat asymmetric hyperintensities in three patients (p5 (Fig. 2b), p6, p7). In the oldest patients they were indistinguishable from white matter changes associated with ageing and arterial hypertension and new lesions in p7 during follow-up could not be differentiated from cerebrovascular changes (Fig. 2g,h). Extent of white matter changes slightly increased over 7 years’ follow-up in p1 (Fig. 2c,d), and was not progressive in p4.

White matter changes were present in only three of eight control patients, all of these high excretors: The youngest (c1, Fig. 2a) had periventricular and the macrocephalic brothers had extensive white matter changes (c6, c7), in the latter not progressive during 8 years’ follow-up (Fig. 2e,f).

### Grey matter changes

No patient had striatal changes typical of GA1. Unilateral gliosis defect and susceptibility artefact of the right anterior caudate and lenticular nucleus in p7 was consistent with postischemic residuum. New cortico-subcortical ischemic lesions as well as stenosis of the left medial cerebral artery developed during follow-up and stenosis of the left medial cerebral artery became apparent on MRI at the age of 70 years, not different from non-GA1 patients with arterial hypertension and atherosclerosis.

Pallidal hyperintensity was observed in two *late-onset* and two high-excreting control patients with additional T2-hyperitensity of the dorsomedial thalamus in one *late-onset* patient (p1; Fig. 2d). The dentate nucleus was T2-hyperintense in five *late-onset* and one high-excreting control patient. In one control patient (c2) slight enlargement of the right dorsal thalamus with focal T2-hyperintensity had been present since the age of 7 months differing by its slight T1-hypointensity from thalamic signal alterations in p1 (Fig. 1c).

Deep grey matter T2-hyperintensity remained unchanged in the two *late-onset* (p1, p4) and the two control patients (c6, c7) with follow-up periods between 7 and 11 years.

### Subependymal lesions

**Fig. 3:**
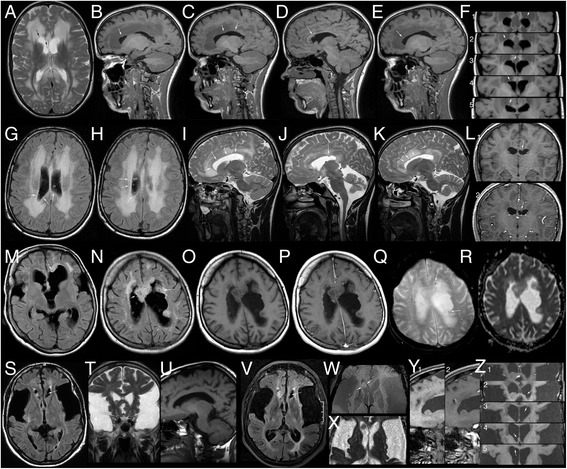
Examples of subependymal lesions. **a**-**f** MRI of *late-onset patient p1* at the age of 15.7 years with the (pre-existing) nodular subependymal lesion in the medial roof of the *right* lateral ventricle (*arrows* in **a**, **d**, **f**_3_) and four incipient lesions in the roof of both lateral ventricles (*arrows* in **a** (lat.), **b** and **f**_2_, in **c** and **f**_3,5_, in **e** and **f**_1_). (**a** *:* T2w; **b** *-* **f** *:* T1w). **g**-**l** MRI of *late-onset patient p2* at 14.7 years with multiple, small, FLAIR-hyperintense nodularities (**g**, **h**, *arrows*) resulting in a “bumpy” surface of the ventricular roof on the T2w sagittal images (**i** *right*, **k** *left* lateral ventricle) and one larger nodule in the roof of the *left* ventricle without enhancement (*arrows* in **j**, **l**_1_, **l**_2_). NB extensive white matter changes. (**g**, **h**: FLAIR; **i**-**k**: T2w; **l**_1_, **l**_,2_: T1w ± GAD). **m**-**r** *Late-onset patient p6* with the most extensive, confluent subependymal lesions with multiple, small cystic areas (hypointense on FLAIR-images **m**, **n**, *arrows* in **n**), facilitated diffusion (**r**), punctate susceptibility artefacts (*arrows* in **q**) and punctate enhancement (compare **p** with **o**). (**m**, **n**: FLAIR; **o**, **p**: T1w ± GAD, **q**: T2*; **r**: ADC). **s**-**z** MRIs of *late-onset patient p7* at the age of 61 (S-U) and 73 years (**v**-**z**). Initially there are two lesions, a small one in the *left* anterior horn (*arrow* in **s**) without significant change on follow-up (*arrows* in **v**, **z**_2_) and a larger one in the *right* anterior horn (**s**, **z**_2_, arrows in **t**, **u**), which increases mildly in size from approx. 10x9x8 mm to 13x9x9 mm during the 12 year period. Punctate susceptibility artefacts (*arrows* in **w**), facilitated diffusion (**x**), and some superficial, linear enhancement (compare **y**_1_ with **y**_2_ and **z**_1_ with **z**_2_) are present, similar to the large lesions in p6. Two additional, small nodules in septal surface of the *right* anterior and the roof of the left horn (*arrows* in **z**_3,4_) have been detectable and without significant changes since MRIs at 65 and 66 years. MRI at 73 years depicts an additional, incipient lesion in the roof of the *right* lateral ventricle (*arrow* in **z**_5_). (**s**, **v**: FLAIR; **t**: T2w; **u**, **y**_1_, **z**_2–5_: T1w, **y**_2_,, **z**_1_: T1w + GAD, **w**: SWI; **x**: ADC)

Subependymal lesions were observed in six *late-onset* patients, namely all but the two maternal GA1 patients, and in one high-excreting control patient (c7). All lesions were located at the border of the lateral ventricles, most commonly the roof, ranging from single, (semi-)nodular lesions to multiple lesion of up to 19 mm diameter (Fig. 3). They were not observed before the age of 12 years.

In the three younger *late-onset* patients diagnosed between eight and 18 years (p1, p2, p5) and in control patient c7 with MRIs at 14–22 years, subependymal lesions presented as small, smooth, round thickenings of the ventricular border of up to 3 mm diameter with isointensity in T2- and T1-weighted images compared to normal white matter. Follow-up MRIs of p1, p7, and c6 suggest slow growth based on lack of significant change of a 1.5 mm seminodular lesion during a three year period in p1, a slight increase from 2 to 3 mm during eight years in c6 and an increase from 10 to 13 mm during 12 years in p7. In p1 four additional, presumably incipient lesions were identified in the last MRI at the age of 15.6 years: Circumscribed thickenings of the ventricular roof, contrasting with the T1-hypointense, T2-hyperintense periventricular white matter changes (Fig. 3a-f). Similar, presumably incipient lesions were also present in p5 as a single and in p2 as multiple, punctate nodularities in the roof of the lateral ventricles (Fig. 3g-l). In p7 four additional lesions were detected during 12 years of follow-up including a punctate, presumably incipient lesion in the roof of the right lateral ventricle at last follow-up (Fig. 3s-z).

In general, lesions in p6-8, who were diagnosed between 25 and 71 years and imaged between 38 and 70 years, were larger with maximum diameters between 13 and 19 mm, a lobulated contour, one to two punctate susceptibility artefacts, and facilitated diffusion on ADC maps. None had decreased diffusion as an indicator of high cellularity. Some large lesions were inhomogeneous with cystic areas with elevated ADC and FLAIR-hypointensity. In addition to linear superficial enhancement consistent with veins in p6 and p7, there was some punctate superficial enhancement not clearly vascular in p6 with the largest lesions.

## Discussion

We systematically analyzed neuroradiological changes, clinical, and biochemical data in eight patients with *late-onset* GA1. The main results of this study are that (1) clinical presentation is non-specific, that all *late-onset* patients were (2) high-excretors, have (3) characteristic frontotemporal hypoplasia, and (4) commonly subependymal lesions seen on cerebral MRI.

### Clinical characteristics of *late-onset* patients

*Late-onset* GA1 was first reported in a 19 year old patient with headache, nystagmus, upward gaze palsy, fine motor disturbances, and leukoencephalopathy [10]. Additional patients diagnosed between the age of eight and 66 years were subsequently published [9–16, 22–27], including two patients [12] and the initial case of p1 [18] previously reported by our group, who are included in this study, and four patients with maternal GA1 [14–16]. Noteworthy, four of these patients had striatal changes on MRI and/or symptoms of a complex dystonic movement disorder thus qualifying them as *insidious-onset* type [10, 25–27]. One patient diagnosed by genetic family counselling was asymptomatic [23]. In three reported patients macrocephaly and developmental delay/learning disability without motor symptoms had already been noticed during infancy and childhood [11, 15] suggesting *late diagnosis* rather than *late onset* in some patients. As in patients with *acute* and *insidious onset*, macrocephaly was not a reliable clinical indicator, being present in only 50% of our *late-onset* patients. In one patient, it remained an isolated clinical finding until the onset of headaches at age 35 years, subsequently followed by tremor and seizures and later dementia (p8, [12]).

Similar to previous reports, clinical manifestation in our patients was non-specific and more severe in older compared to younger patients: While our oldest patients in and above the 7^th^ decade had clear neurologic signs and symptoms, younger patients were either asymptomatic females identified via their children or predominantly presented with headache, vertigo and nausea. These are common, non-specific, but not necessarily neurologic symptoms which in our experience are sometimes described by GA1 patients after excessive protein intake.

Effects of age at diagnosis on clinical manifestation are difficult to determine since our control patients were no older than 22 years at last follow-up. Three of the six *late-onset* patients diagnosed before the age of 30 years had (non-specific) symptoms compared to only one of eight control patients, which suggests a higher probability of symptoms in *late-onset* patients. This observation is limited by the small number of patients and may result from early treatment of control patients compared to *late-onset* patients.

### MRI in *late-onset* patients

1. Frontotemporal hypoplasia
  Enlarged anterior temporal CSF spaces, incomplete opercularization, and widening of the Sylvian Fissure are the most common and characteristic imaging finding in GA1. They are already present at birth in preterm and term babies and can even be observed during the last trimester of pregnancy [18, 28–30]. The appearance of the anterior temporal lobe and Sylvian fissure in infants is similar to stages of normal development, gyration and opercularization occurring latest in the fronttemporal area. Moreover CSF spaces have been observed to normalize in GA1 patients with early diagnosis and treatment [18, 19, 31–33], for which reason widened anterior temporal and Sylvian CSF spaces in GA1 are consistent with arrested development of the anterior temporal and frontal lobes, namely frontotemporal hypoplasia, but not with atrophy, as there is primarily no irreversible loss of tissue.
  Similar to patients diagnosed in infancy and childhood, frontotemporal hypoplasia is the most common and characteristic imaging finding in *late-onset* patients. Frontotemporal hypoplasia persisted in our three *late-onset* patients over follow-up periods between 7 and 12 years suggesting that normalization is only possible up to a certain period of time.
2. White matter changes
  White matter changes were the most striking imaging finding in the first patient reported as *late-onset* and have been reported for all *late-onset* patients [9–13, 22–27]. They are also frequent in patients with *acute* or *insidious-onset* type, apparently increasing with age [18]. Consistent with the literature, they were present in all our *late-onset* patients but - being non-specific - they are not decisive for the diagnosis of *late-onset* patients. Though more frequent in *late-onset* compared to control patients they do not allow differentiation from either early diagnosed and treated control patients or from patients with *acute* or *insidious-onset* type.
3. Subependymal lesions
  Subependymal mass lesions have been reported in three *late-onset* patients aged 30 to 56 years [13, 22, 23]. In our cohort, subependymal lesions were detected in six of eight *late-onset* patients - all but the two maternal GA1 patients - and in one control patient (c7). In spite of our small study group this high frequency suggests that subependymal lesions are common in *late-onset* GA1 and that they may also occur in adolescent patients treated since childhood. Lesion growth as seen in our three *late-onset* patients with follow-up MRIs appears to be slow. Lesion number and size apparently increase with age and might moreover be related to age at diagnosis.
  Similar to reported patients, location of lesions was exclusive to the lateral ventricles with a predilection for the roof. Location, slow growth, and signal would be consistent with subependymoma. Though somewhat reminiscent of the subependymal hamartomas and subependymal giant astrocytomas of tuberous sclerosis, subependymal lesions of GA1 lack the predilection for the caudothalamic groove. As yet, histopathology has not been reported. With regard to pathogenesis subependymal lesions might reflect distribution of the neurotoxic metabolites GA and 3-OH-GA and/or brain susceptibility, as GA and 3-OH-GA accumulate within the brain due to restricted flux across the blood–brain barrier [34, 35].

### *Late-onset* GA1 - a variant with solely chronic neurotoxicity in patients with a high-excreting phenotype

During the last years, the terms *insidious* and *late-onset* have been used for patients whose clinical manifestation differs from *acute-onset* type GA1. Patients with *insidious-onset* develop a complex dystonic movement disorder due to striatal injury but *without* clinically apparent precipitating event, hereby differing from patients with *acute-onset* [36], while the term *late-onset* has been proposed for patients presenting after the age of six years. It is currently not clear whether or not *late-onset* patients represent a distinctive disease variant, in particular since some reported patients had striatal changes on MRI and/or signs of a dystonic movement disorder thus fulfilling the criteria for *insidious-onset* type GA1 [10, 25–27]. We therefore only included patients without either clinical or MRI evidence of striatal lesions in our study, namely those patients who - though untreated - did not suffer striatal injury despite various infections and other potential triggers during infancy and childhood.

Absence of clinical or MR evidence of striatal injury is a feature shared by both, our *late-on*set and early treated control patients. However, while both groups are exposed to chronic neurotoxicity, cumulative neurotoxicity should be less in control patients due to early start of metabolic treatment, since metabolic treatment is aimed at reducing the intracerebral accumulation of the neurotoxic metabolites GA and 3-OH-GA. Proof of concept has been demonstrated in *Gcdh* ^*−/−*^ *mice* [37]. Consequently, greater cumulative neurotoxicity might explain the (non-specific) symptoms in three of the six *late-onset* patients diagnosed between the age of 8.5 and 29 years compared to one of eight control patients with last follow up between the ages of 9 and 26 years.

Treatment however does not preclude development or progression of MR changes in *late-onset* or control patients, e.g. white matter changes were slightly progressive in *late-onset* patient p1 despite initiation of treatment and resolution of clinical symptoms and developed in control patient c5 between MRIs at 4 and 11 years (for initial findings which have been previously been reported by us see case 4 in [18]). Another example is the detection of subependymal nodules in *late-onset* patient p1 and control patient c7 after initiation of treatment.

The exclusively high excreting phenotype of *late-onset* patients in our study was unexpected, since the proportion is approximately two thirds in infants and children with GA1 [2, 38]. A correlation of genotype with biochemical phenotype has been reported in GA1, whereas as yet no correlation of either genotype or biochemical phenotype with clinical phenotype is known [2, 38, 39]. High excretors show a complete loss of GCDH activity in contrast to up to 30% residual enzyme capacity in low excretors who predominantly carry missense mutations on at least one GCDH allele [5, 39]. Without treatment high- and low-excretors have a similar risk of developing striatal damage and dystonia which determine clinical outcome [2]. This has been explained by entrapment of neurotoxic metabolites in the brain compartment and, subsequently, high cerebral concentrations of GA and 3-OH-GA in both, low- and high-excreting patients [34, 35].

However, several observations challenge this view and indicate an increasing influence of the biochemical phenotype with growing age: All our *late-onset* patients are high excretors and no low-excreting *late-onset* patient has been reported. In addition, MRI was normal in all four low-excreting control patients, but in none of the high-excreting control patients. This finding, however, is limited by the small number of control patients. In addition, we have recently unravelled the high excreting phenotype to be a risk factor for increased chronic cerebral GA accumulation and progressive neuroaxonal compromise in high-excretors compared to low excretors [40]. We therefore hypothesize that the biochemical phenotype co-determines whether or not clinical signs and symptoms will occur in potential *late-onset* patients and that this is related to higher degree of chronic neurotoxicity with greater intracerebral accumulation of GA and 3-OH-GA in high excretors. Further studies are necessary to evaluate differences in the clinical course in both biochemical subgroups and to elucidate the impact of MR changes on clinical phenotype.

## Conclusion

While clinical manifestation is non-specific in *late-on*set GA1, frontotemporal hypoplasia in particular if in combination with subependymal nodules is characteristic of late-onset GA1 and should trigger investigation for GA1.

Apart from their apparent non-susceptibility for striatal injury despite lack of treatment, patients with *late-on*s*et* GA1 are not categorically different from early treated control patients. We hypothesize that “*late-onset* GA1” is the result of long-term toxicity in untreated GA1 patients with a high excreting phenotype. Accordingly, differences between “*late onset* GA1” and early treated patients might reflect the higher long-term risk of high excretors compared to low excretors and of individuals remaining undiagnosed and untreated for years, even decades. As long-term clinical outcome in GA1 is unclear, implementation of this observation for clinical management and monitoring remains to be elucidated.

## Additional file

Additional file 1: Case report C7. (DOC 27 kb)
