# Supplementary material for: Extrastriatal changes in patients with late-onset glutaric aciduria type I highlight the risk of long-term neurotoxicity
Source: Orphanet J Rare Dis. 2017 Apr 24;12:77. doi: 10.1186/s13023-017-0612-6 (PMC5402644; doi:10.1186/s13023-017-0612-6)
Supplement: Additional file 1: — Case report C7. (DOC 27 kb) [file 13023_2017_612_MOESM1_ESM.doc]

**Supplemental material 1**

**Case report c7:**

Patient 7, born in 1943, has an unremarkable family history and development. He experienced various febrile infections and episodes of vomiting during childhood but without encephalopathic crisis or subsequent dystonic movement disorder. Prostate cancer (T1c, Gleason 6, malignity grade 2a), was diagnosed at age 69 years and, due to assessment as low-risk, a watch-and-wait strategy was adopted.

He first came to neurological attention at the age of 62 years when he was admitted to a peripheral hospital with acute onset of double vision, dysarthria, gait disturbance and thermhypesthesia of his left hand. Clinical examination additionally revealed tongue deviation to the right, mild intention tremor, and lower facial nerve asymmetry. MRI revealed ischemia of the right caudal dorsolateral medulla oblongata as well as an old defect of the right anterior lenticular and caudate nucleus, patchy white matter hyperintensities and wide frontotemporal CSF spaces. As screening for vascular risk factors had identified an increased concentration of homocysteine and treatment with folinic acid, vitamin B complex and dihydropyridamol was started. One week later gait, vision, and articulation as well as the initially observed mild focal EEG abnormalities had normalized. Peripheral electrophysiology results were consistent with polyneuropathy.

At the age of 65 years he suffered an episode of acute confusion and memory deficits. On clinical examination a residual right-sided hemiparesis was noted. Compared to the previous MRI white matter changes were reported to have slightly increased and the “bilaterally nearly absent opercularization” was mentioned as unchanged. In addition a small, new, postischemic defect of the right medial temporo-occipital gyrus was depicted. Due to a positive borrelia IgM titer in serum he was treated with ceftriaxon for 14 days. While CSF protein was massively increased, there was no intrathecal antibody production. Thromboembolic screening was again negative. Arterial hypertension was newly diagnosed and treatment with ramiprile, bisoprolole, and hydrochlorothiazide initiated.

He was admitted again in the following year with cognitive deficits and another four years later, at the age of 70 years, with acute onset of confusion, aphasia and apraxia. Clinically severe dehydration and residual right-sided hemiparesis were noted, however with signs of acute or subacute stroke on MRI. The bilaterally nearly absent opercularization was mentioned again and MRI moreover depicts new, postischemic gliosis of the left median occipital lobe as well as stenosis of the left medial cerebral artery. CSF protein continued to be elevated. Following rehydration and discontinuation of baclofen (started for neck pain a few days earlier), the acute symptoms completely resolved.

In March of the following year, at the age of 71 years, he was admitted to a psychiatric department of his local hospital for evaluation of increasing cognitive deficits, confusion, forgetfulness, aphasia, and episodes of mental absence first recognized by his wife. His general condition and physical examination were normal. Psychiatric assessment revealed deficits in temporal orientation (other qualities of orientation normal), mood swings with depressive and aggressive episodes, lethargy, and, deficits of (primarily short) memory function with no signs of personality disorder. Re-evaluation of cerebral MRIs depicting the characteristic frontotemporal hypoplasia led to diagnostic investigation for GA1. Urinary concentration of GA was highly elevated (2143 mmol/mol creatine), while concentration of 3-OH-GA was not recorded. Diagnosis was genetically confirmed by detection of homozygous mutation p.Arg402Trp in *GCDH* gene. Metabolic treatment with oral carnitine supplementation and a protein controlled diet (natural protein 40g/day) using natural protein with low lysine content and avoiding lysine-rich food were initiated. In addition, his confusional state and absence episodes were classified as non-convulsive seizures although EEG showed no specific epileptic activity. He therefore was started on levetiracetame (LVT). In the following months his mental state deteriorated with increasing lethargy and confusion and he started to receive day-care several times a week.

In December of the same year, at age 71, he was referred to our outpatient metabolic clinic years for continuing deterioration with progressive dementia and cognitive deficits. Neurologic exam revealed reduced strength in his left upper extremity, fine motor and coordination deficits and tremor but no signs of dystonia or chorea. Biochemical work-up still showed high excreting phenotype and markedly elevated 3-OH-GA (Table 1). Neuropsychological evaluation showed mild dementia (22/30 points in mini-mental status test) as well as a total IQ of 66 with deficits primarily in perceptive logical thinking, as assessed by Wechsler Adult Intelligence Scale (WAIS-IV). Rey-Osterrieth Complex Figure Test showed normal visuomotoric functions.

Six months later, at the age of 72 years, he was again admitted to the local neurology department with acute aphasia and apraxia in the context of dehydration. Symptoms resolved with rehydration. A new, demarcated defect of the left occipital lobe was reported on MRI, but no acute lesions with restricted diffusion were present. EEG did not show signs of a non convulsive status epilepticus. However, LVT was increased and clobazam was started. Cognitive decline as reported by his wife was progressing.

He first suffered non-convulsive generalized seizures at the age of 73 years. Dementia had further progressed and he was by then living in a nursing home. He has recently been reassessed at our outpatient metabolic clinic. He was in a calm mood, orientated with respect to his person, but not orientated in time or space. Dementia had progressed and frequency of seizures with aphasia and absence episodes increased to monthly. Tremor had also progressed and use of hands been reduced to a minimum. For several months he has been using a walker-rollator. He now needs assistance for every daily situation. Urinary and rectal incontinence have developed and he has lost 18 kg body weight since last follow-up at our department two years ago. MRI revealed a new postischemic residuum in the left parietal border zone compared to the previous MRI in addition to the pre-existing residua in both occipital lobes and in the right anterior lenticular and caudate nucleus. Reviewing MRIs performed between the age of 61 and 73 years, white matter changes were progressive and there was moderate, generalized volume loss with widening of ventricles and sulci over time (Fig. 2G, H), while frontotemporal hypoplasia was stable (Fig. 1M-R). Subependymal lesions were present from the beginning with a slight increase of the largest lesions in the right anterior horn from approximately 10x9x8 mm to 13x9x9 mm. A small seminodule in the caudal left anterior horn was also detectable since the initial MRI at the age of 61 years, two more small lesions became visible at the septal surface of the right anterior horn (65 years) and in the roof of the left anterior lateral ventricle (66 years), these without unequivocal increase. With a smaller minimal slice thickness (1 mm) the current MRI additionally depicts a presumably incipient lesion in the roof of the right anterior horn (Fig. 3S-Z).
